# Screening for Tuberculosis in Health Care Workers: Experience in an Italian Teaching Hospital

**DOI:** 10.1155/2017/7538037

**Published:** 2017-02-27

**Authors:** Christian Napoli, Filippo Ferretti, Filippo Di Ninno, Riccardo Orioli, Alessandra Marani, Maria Giuditta Sarlo, Claudio Prestigiacomo, Assunta De Luca, Giovanni Battista Orsi

**Affiliations:** ^1^Department of Medical Surgical Sciences and Translational Medicine, “Sapienza” University of Rome, Via di Grottarossa 1035/1039, 00189 Rome, Italy; ^2^Health Direction, University Hospital Sant'Andrea, Via di Grottarossa 1035/1039, 00189 Rome, Italy; ^3^Department of Public Health and Infectious Diseases, “Sapienza” University of Rome, Piazzale Aldo Moro 5, 00185 Rome, Italy

## Abstract

Health care workers (HCW) are particularly at risk of acquiring tuberculosis (TB), even in countries with low TB incidence. Therefore, TB screening in HCW is a useful prevention strategy in countries with both low and high TB incidence. Tuberculin skin test (TST) is widely used although it suffers of low specificity; on the contrary, the in vitro enzyme immunoassay tests (IGRA) show superior specificity and sensitivity but are more expensive. The present study reports the results of a three-year TB surveillance among HCW in a large teaching hospital in Rome, using TST (by standard Mantoux technique) and IGRA (by QuantiFERON-TB) as first- and second-level screening tests, respectively. Out of 2290 HCW enrolled, 141 (6.1%) had a positive TST; among them, 99 (70.2%) underwent the IGRA and 16 tested positive (16.1%). The frequency of HCW tested positive for TB seems not far from other experiences in low incidence countries. Our results confirm the higher specificity of IGRA, but, due to its higher cost, TST can be considered a good first level screening test, whose positive results should be further confirmed by IGRA before the patients undergo X-ray diagnosis and/or chemotherapy.

## 1. Introduction

Worldwide, 9.6 million people are estimated to have fallen ill with tuberculosis (TB) in 2014 [1]. Moreover, latent TB infection (LTBI) global burden is regularly reported to be approximately one-third of world population [1]. Since people with LTBI are at risk for developing active TB in their lifetime, the World Health Organization (WHO) developed a guidance for managing LTBI, reporting evidence on testing and treatment [2].

With TB incidence rate being considerably below 10 cases per 100,000 inhabitants over the last 10 years, Italy is considered a low-burden country, where specific population subgroups are affected [3].

Health care workers (HCW) are particularly at risk of acquiring TB in all its forms [4, 5] even in countries with low TB incidence, such as Italy.

A meta-analysis estimated that the average annual risk for developing TB disease was threefold higher for HCW (across all settings) compared to the general population [5]. Also, multidrug-resistant TB (MDR-TB) strains are associated with diagnosis delay, less effective treatment, and longer contact periods with infectious patients, increasing their potential transmission to HCW. Consequently, HCW are up to six times more likely to be hospitalized for MDR-TB than the population they care for [6]. Similarly, health care students may present a higher TB infection risk than ordinary population [7].

Therefore, prevention in health care settings is particularly important [1, 8, 9], and TB screening aimed at early identification of cases is the principal step both in countries with low and high TB incidence [10, 11]. Also WHO guidelines strongly recommend a systematic testing for HCW [2].

With regard to HCW, scientific evidence underlines preemployment and routine (e.g., annual, biannual etc.) screening importance, depending on risk assessment even in low incidence countries [12]. Traditionally, tuberculin skin test (TST) has been used for HCW TB screening; however its low specificity among BCG-vaccinated HCW and the boosting phenomenon of repeated tuberculin skin test may provide false positive results, with a potential negative consequence of unnecessary chest X rays and/or isoniazid prophylaxis. In vitro enzyme immunoassay tests based on interferon-gamma released (IGRAs) quantification have demonstrated superior specificity and sensitivity compared with TST and, moreover, only one visit is required [13–15]. In particular, QuantiFERON-TB test in HCW has excellent utility and accuracy especially in BCG-vaccinated populations in low incidence countries [16], but the test is greatly expensive. In Italy, the latest TB prevention national guidelines for HCW launched by the Ministry of Health indicate TST as the first-level screening test. When TST proves positive, then it may be confirmed by the IGRAs [17].

The present study reports the results of a three-year TB surveillance among HCW in a large Italian teaching hospital.

## 2. Materials and Methods

The study was carried out during the 2013–2015 period, in the 450-bed teaching hospital “Sant'Andrea” in Rome, a tertiary referral centre with approximately 24.000 inpatient discharges per year. Overall, the hospital accounts for about 1,800 HCW, distributed in different working categories.

According to an internal procedure, all HCW undergo LTBI screening both when they start working and at any time of their working life with a frequency based on their infection risk. To this aim, after an occupational medicine audit, HCW accessed an outpatient clinic, where TST was performed by two trained nurses using the standard Mantoux technique, consisting of an intradermal injection of 0.1 ml (5 IU) of* Mycobacterium tuberculosis* purified proteins (PPD). The test was read by measuring the skin reaction 72 hours after the PPD injection. In accordance with the Italian guidelines [17] a skin reaction ≥ 10 mm was considered positive. During the three-year study period different commercial tuberculin products were used. During the three-year study period, different commercial tuberculin products were used: from January 2013 to July 2014, “PPD tuberculin mammalian” (produced by BB-NCIPD Ltd.); from August 2014 to July 2015, “Tubertest” (produced by Sanofi Pasteur MSD); and from August to December 2015, “PPD tuberculin mammalian” once again.

Since May 2014, the QuantiFERON-TB Gold (QFT®) was introduced in the hospital routine TB surveillance as a second-level screening test. The test was considered positive when ≥0.35 IU/ml.

Data regarding all TB screening tests are routinely stored into a password-protected database, that was anonymously interrogated in order to carry out a descriptive study on positive tests prevalence between January 2013 and December 2015. Risk factors for TB, both at and outside work, were also analyzed and their association with TST and IGRA test positivity was evaluated.

In order to acquire additional information on TB positivity distribution in our hospital two further databases were interrogated. The first one was the mandatory reporting of infectious diseases. All TB cases diagnosed in the enrolled hospital and reported to the local health authorities were analyzed. The second data source was the Hospital Discharge Database (HDD), which contains anonymized administrative and health data regarding discharged patients, which all public and private hospitals are legally required to report. For each patient, the main discharge diagnosis represents the clinical condition which took up the greatest amount of resources and involved the greatest cost for the hospital. Diagnoses were coded using the nomenclature of the International Classification of Diseases, 9th revision, Clinical Modification (ICD-9-CM). We collected and examined data regarding TB hospitalizations for the period 2013–2015, considering ICD-9-CM codes from 0.11 to 0.18. Multiple hospital admissions for the same case were identified by a unique key and excluded from the analysis.

The *chi*^2^ statistical test was used to evaluate the association between independent variables. A value of *p* < 0.05 was considered significant. Adjusted odds ratios (OR) and 95% confidence intervals (CI) were calculated for risk factors for a positive TST and QFT using a multivariate logistic regression model. Finally, in order to evaluate a difference in terms of positive results between* PPD tuberculin mammalian* and* Tubertest*, the* two-sample Z-Test* was used.

All statistical analyses were performed using SPSS software version 14.

## 3. Results

Overall, 2,290 HCW (913 men and 1,377 women) underwent TB screening by TST between January 2013 and December 2015. HCW median age was 44 ± 13 years and most represented professional categories were physicians and nurses, principally belonging to medical wards. Sample distribution by gender, TB exposure at work, vaccination status, working area, and professional category is shown in Table 1. All enrolled HCW were born in EU/EEA countries, except one. None of the enrolled HCW reported TB exposure outside work or a TB diagnosis history.

**Figure 1:**
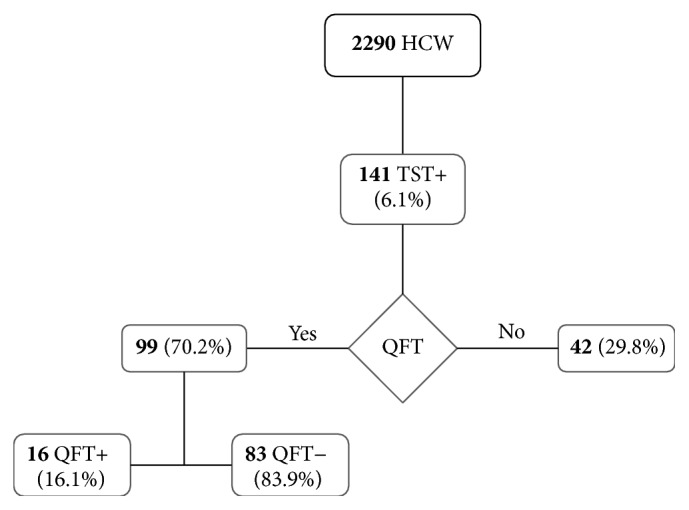
Flowchart: study population, TST, and IGRA results.

Overall, 141 (6.1%) HCW had a positive TST (Figure 1), with a skin reaction median value of 15 mm (interquartile range ±8,5). Table 1 shows the percentage of TST positivity stratified for the above-mentioned characteristics. The percentage of TST positive HCW was higher in 2015 (Table 2). The results stratified by TST commercial products are reported in Table 3.

Among HCW with a positive TST, 99 (70.2%) underwent the second-level screening test (QFT), whereas the remaining 42 HCW were not tested by IGRA since it was introduced in the hospital procedure only from May 2014. Out of 99 HCW screened by IGRA, 16 (16.1%) tested positive (Figure 1); the median QFT value was 5.5 IU/ml (interquantile range ±2.1). The percentage of positive IGRA tests was 12.8% (5/39) and 18.3% (11/60) in HCW with positive TST in 2014 and 2015, respectively.

Both TST and IGRA positive tests were not statistically associated with the working area (*p* > 0.05). TST and IGRA positive HCW were born in Italy and reported a clear previous TB exposure at work in 55 cases (39%) and 11 (68.8%), respectively (Table 1). When considered as independent variables, a TB exposure at work was significantly associated with TST and IGRA positivity (*p* < 0.001 and *p* < 0.05 respectively). Among HCW tested positive for TST, 90 (63,8%) reported to have been vaccinated for a* Bacille Calmette-Guérin* (BCG), of which 4 were confirmed by IGRA (Table 1). When considered as independent variables, to be vaccinated was associated with TST positivity (*p* < 0.05), but not with IGRA positivity (*p* > 0.05). The multivariate regression model showed that the statistically significant risk factors for a positive TST were a TB exposure at work and to be vaccinated for BCG; on the contrary, to be vaccinated was a protective factor for a positive IGRA (Table 1).

Comparing the TST positivity in 2015 to those in 2013 and 2014 (Table 2), the differences were statistically significant (*p* < 0.05). The difference in terms of positive results between the two commercially available tuberculin skin tests used* (PPD tuberculin mammalian* and* Tubertest)* was not statistically significant (*p* < 0.05). Moreover, also the rates of positive IGRA tests increased in 2015, although this difference was not statistically significant (*p* > 0.05). Therefore, the increased TST positivity in 2015 was considered genuine and not due to the test used.

The hospital infectious disease notification database was also consulted to verify a possible increase in TB cases among patients or workers, but the number of reported TB cases decreased in 2015 (Table 4). The same evidence was drawn from the HDD analysis, as the number of cases with primary diagnosis of TB decreased from 70 in 2014 to 38 in 2015 and the same was observed for the secondary diagnosis of TB that also decreased from 17 in 2014 to 11 in 2015.

## 4. Discussion

The present study reports the results of a two-step TB screening system carried out in HCW of an Italian teaching hospital. It shows a rate of TB infections at three years with TST and at twenty months with IGRA of 6.1% and 1.58%, respectively.

The annual TST positivity prevalence ranged between 5.03% and 8.34%. These data are higher if compared to the results of an extensive review showing that median annual estimates of latent TB in HCW are expected to range from 3.8% to 6.9% and 8.4% in low, intermediate, and high incidence country, respectively [5]. However, other studies have documented an annual TST conversion rate ranging from 1% up to 10% among exposed HCW [18–21]. It should also be noted that in all these studies authors reported the incidence, defined as tuberculin conversion after a documented negative-baseline TST. Differently, in our hospital, we documented the results of a single test; therefore, no incidence was evaluable.

A study conducted in three Norwegian hospitals showed that, out of 387 HCW investigated, 55.3% were considered positive at TST, of which 4.7% were confirmed by IGRA. The percentage of TST positive was greatly higher than in our experience and, on the contrary, the percentage of IGRA positive seems lower than our results (16.1%); however, it should be noted that the authors considered the TST positive when the skin reaction was ≥ 6 mm [22], whereas in our study the cut off was 10 mm.

Our TST results were in line with another two-step TB screening in Italy that reported a 6.7% TST positive rate, although the second step IGRA positive rate was 25% [23].

Our study demonstrated that, although there is an association between the risks of a positive TST or IGRA with a working exposure, this risk was not associable with working in high-risk areas; as previously described, some authors found an association with working in wards, as emergency department, where undiagnosed patients are seen [24].

Also the professional category was not associated with a higher risk of TST of IGRA positivity, in our study. The administrative staff showed the highest percentage of positive TST (9,5%), but this data seems unreliable, since the number of screened workers is not representative of the whole area; moreover, none of TST positive administrative staff was confirmed at IGRA. In any case, it should be noted that an increased risk of IGRA positivity was also reported in the administrative staff during a TB screening experience among HCW in Germany [25].

No association was studied between country of birth (used as proxy of country of origin) and screening positivity since the TST positive HCW were all born in Italy. This is not consistent with data reported at EU level: the countries reporting most TB cases originating from other EU/EEA were Germany and Italy [26].

We observed an increased TST positivity in HCW during the last year of surveillance; the analysis excluded possible bias due to different commercial tuberculin products adopted along the three years. Moreover TST results were consistent with the higher positivity reached during 2015 by the IGRA tests. In our opinion it might be possible that immigration may had an impact on this increased prevalence as suggested by other authors [27]. In our hospital the number of immigrants increased in 2015, given the existence of a referral out-patient-clinic of infectious disease for a large immigration centre located in the same area of the hospital. The other data sources did not confirm an increasing trend in TB cases, but these data do not include information regarding infectious diseases in irregular migrants and, moreover, those surveillance systems are affected by under- or misreporting [28]. In any case, no data are available from the present study in order to confirm this hypothesis; therefore, caution must be paid before generalizing this interpretation.

With regard to the test used for TB screening, different limits of TST have been described, as low specificity, but on the other hand it is easy to use and not expensive. In our experience TST allows highly reducing the number of people that should undergo more expensive and dangerous tests, confirming that IGRA performed after a positive TST is more cost effective than IGRA as first screening, especially in low incidence countries [13].

Moreover, a large part of TST positive HCW had an history of BCG vaccination (90/141), while the great majority of IGRA positive (12/16) were not vaccinated; this is consistent with data that underline false TST positivity in BCG-vaccinated populations [16, 29].

Some limitations of the study include that we did not have a negative TST baseline; therefore, no incidence is available. Moreover, in our study, the TST was performed only to those HCW who started working and/or were considered at risk for TB during the three-year period; therefore, since not all the personnel population was tested, it is not possible to exclude a selection bias. Finally, a precise identification of possible exposure outside work was not possible, as the occasion of contact with TB is difficult to identify, and since the data analyzed were anonymized, no other risk factors such as the socioeconomic status have been investigated.

## 5. Conclusions

In our experience, frequency of HCW tested positive for TB seems low and, although different study protocols make it hard to compare data, our results are not so far from other experiences in low incidence countries.

The study results strongly support that, in accordance with the Italian guidelines [17], the TST use as first step screening may be recommended, in particular for those HCW undergoing serial testing. At the same time, the IGRA central role as TB second step screening in order to reduce the number of people that should undergo more invasive tests is confirmed, especially in those countries with high BCG vaccination rate.

Finally, our experience did not show any significant difference between different commercial products for TST execution, but, since the study was a retrospective study over a three-year follow-up period, further ad hoc studies are necessary for confirming this data.

## Figures and Tables

**Table 1:** Characteristics of the study population including frequencies and the results of logistic regressions.

Characteristics	Enrolled HCW *N* (%)	TST positive HCW *N* (%)	OR^*∗*^	95% CI	*p* value	IGRA positive HCW^*∗∗∗*^ *N* (%)	OR^*∗∗*^	95% CI	*p* value
*Gender*									
Males	913 (39.9)	58 (41.1)	1	—	—	9 (56.3)	1	—	—
Females	1377 (60.1)	83 (58.9)	0.83	0.57–1.19	0.306	7 (43.7)	0.29	0.07–1.12	0.072
*TB exposure at work*									
No	1968 (85.9)	86 (61.0)	1	—	—	5 (31.3)	1	—	—
Yes	322 (14.1)	55 (39.0)	4.5	3.11–6.49	<0.001	11 (68.7)	3.34	0.95–11.75	0.060
*BCG vaccination*									
No	1087 (47.5)	51 (36.2)	1	—	—	12 (75.0)	1	—	—
Yes	1203 (52.5)	90 (63.8)	1.68	1.17–2.42	0.005	4 (25.0)	0.22	0.06–0.84	0.028
*Working areas*									
Surgical wards	531 (23.2)	35 (24.8)	1	—	—	5 (31.3)	1	—	—
Medical wards	843 (36.8)	53 (37.6)	1.03	0.65–1.62	0.905	7 (43.7)	0.39	0.06–2.80	0.352
Services	504 (22.0)	24 (17.0)	0.88	0.49–1.59	0.681	2 (12.5)	0.91	0.22–3.85	0.903
Emergency, ICU	412 (18.0)	29 (20.6)	1.01	0.60–1.71	0.963	2 (12.5)	0.32	0.04–2.52	0.276
*Professional category*									
Administrative staff	42 (1.8)	4 (2,9)	1	—	—	0	—	—	—
Medical doctors	923 (40.3)	46 (32.6)	0.52	0.17–1.57	0.245	8 (50.0)	—	—	—
Technicians	180 (7.9)	5 (3.5)	0.33	0.08–1.35	0.124	0	—	—	—
Auxiliary, cleaning staff	201 (8.8)	7 (5.0)	0.35	0.09–1.29	0.114	0	—	—	—
Nurses	927 (40.5)	79 (56.0)	0.91	0.30–2.71	0.859	8 (50.0)	—	—	—
Nonmedical Doctors (biologists, pharmacists)	17 (0.7)	0	—	—	—	—	—	—	—

*Total*	*2290*	*141*				*16*			

^*∗*^The final multivariate logistics model includes the variables gender, TB exposure at work, BCG vaccination, working areas, and professional category.

^*∗∗*^The final multivariate logistics model includes the variables gender, TB exposure at work, BCG vaccination, and working areas.

^*∗∗∗*^This data refers to the period of May–December 2014.

**Table 2:** Number of workers tested for TB by TST, stratified by year and results.

	2013	2014	2015	Tot
Negative N. (%)	661 (94.97)	829 (94.97)	659 (91.66)	2149 (93.84)
Positive N. (%)	35 (5.03)	46 (5.26)	60 (8.34)	141 (6.16)

Tot	**696**	**875**	**719**	**2290**

**Table 3:** Number of workers tested for TB by TST and IGRA, stratified by TST test.

Period	TST used	% TST positive (*n*/*N*)	% IGRA positive (*n*/*N*)
January 2013–July 2014	PPD tuberculin mammalian	5.3 (66/1250)	—
August–December 2014	Tubertest	4.6 (15/321)	12.8% (5/39)^*∗*^
January–July 2015	Tubertest	7.3% (33/450)	18.1% (6/33)
August–December 2015	PPD tuberculin mammalian	10.4% (27/269)	18.5% (5/27)

^*∗*^This data refers to the period of May–December 2014.

**Table 4:** Number of TB reported cases, in 2013–2015.

	2013	2014	2015
TB notifications	32	22	16
TB primary diagnosis (HDD)	57	70	38
TB secondary diagnosis (HDD)	17	17	11
